# Mapping digital health ecosystems in Africa in the context of endemic infectious and non-communicable diseases

**DOI:** 10.1038/s41746-023-00839-2

**Published:** 2023-05-26

**Authors:** Tsegahun Manyazewal, Mohammed K. Ali, Tedla Kebede, Matthew J. Magee, Tewodros Getinet, Shivani A. Patel, Damen Hailemariam, Cam Escoffery, Yimtubezinash Woldeamanuel, Nardos Makonnen, Samrawit Solomon, Wondwossen Amogne, Vincent C. Marconi, Abebaw Fekadu

**Affiliations:** 1 Addis Ababa University, College of Health Sciences, Center for Innovative Drug Development and Therapeutic Trials for Africa (CDT-Africa), Addis Ababa, Ethiopia; 2 Emory University, Rollins School of Public Health, Hubert Department of Global Health, Atlanta, GA USA; 3 Emory University, School of Medicine, Department of Family and Preventive Medicine, Atlanta, GA USA; 4 Addis Ababa University, College of Health Sciences, School of Medicine, Addis Ababa, Ethiopia; 5 St. Paul’s Hospital Millennium Medical College, School of Public Health, Addis Ababa, Ethiopia; 6 Addis Ababa University, College of Health Sciences, School of Public Health, Addis Ababa, Ethiopia; 7 Emory University, Rollins School of Public Health, Department of Behavioral, Social, and Health Education Sciences, Atlanta, GA USA; 8 University of Virginia, School of Medicine, Department of Emergency Medicine, Charlottesville, VA USA; 9 Addis Ababa University, College of Health Sciences, Addis Ababa, Ethiopia; 10 Emory University School of Medicine and Rollins School of Public Health, Atlanta, GA USA

**Keywords:** Infectious diseases, Cardiovascular diseases

## Abstract

Investments in digital health technologies such as artificial intelligence, wearable devices, and telemedicine may support Africa achieve United Nations (UN) Sustainable Development Goal for Health by 2030. We aimed to characterize and map digital health ecosystems of all 54 countries in Africa in the context of endemic infectious and non-communicable diseases (ID and NCD). We performed a cross-national ecological analysis of digital health ecosystems using 20-year data from the World Bank, UN Economic Commission for Africa, World Health Organization, and Joint UN Programme on HIV/AIDS. Spearman’s rank correlation coefficients were used to characterize ecological correlations between exposure (technology characteristics) and outcome (IDs and NCDs incidence/mortality) variables. Weighted linear combination model was used as the decision rule, combining disease burden, technology access, and economy, to explain, rank, and map digital health ecosystems of a given country. The perspective of our analysis was to support government decision-making. The 20-year trend showed that technology characteristics have been steadily growing in Africa, including internet access, mobile cellular and fixed broadband subscriptions, high-technology manufacturing, GDP per capita, and adult literacy, while many countries have been overwhelmed by a double burden of IDs and NCDs. Inverse correlations exist between technology characteristics and ID burdens, such as fixed broadband subscription and incidence of tuberculosis and malaria, or GDP per capita and incidence of tuberculosis and malaria. Based on our models, countries that should prioritize digital health investments were South Africa, Nigeria, and Tanzania for HIV; Nigeria, South Africa, and Democratic Republic of the Congo (DROC) for tuberculosis; DROC, Nigeria, and Uganda for malaria; and Egypt, Nigeria, and Ethiopia for endemic NCDs including diabetes, cardiovascular disease, respiratory diseases, and malignancies. Countries such as Kenya, Ethiopia, Zambia, Zimbabwe, Angola, and Mozambique were also highly affected by endemic IDs. By mapping digital health ecosystems in Africa, this study provides strategic guidance about where governments should prioritize digital health technology investments that require preliminary analysis of country-specific contexts to bring about sustainable health and economic returns. Building digital infrastructure should be a key part of economic development programs in countries with high disease burdens to ensure more equitable health outcomes. Though infrastructure developments alongside digital health technologies are the responsibility of governments, global health initiatives can cultivate digital health interventions substantially by bridging knowledge and investment gaps, both through technology transfer for local production and negotiation of prices for large-scale deployment of the most impactful digital health technologies.

## Introduction

The United Nations (UN) Sustainable Development Goal 3 (SDG 3) has been in effect since 2015 in response to the demand for ensuring healthy lives and promoting well-being for everyone by 2030, where endemic infectious and non-communicable diseases (IDs and NCDs) hold top priority. SDG 3 includes a specific target for endemic IDs (SDG 3.3) that anticipates ending by 2030 the epidemics of AIDS (SDG 3.3.1), tuberculosis (SDG 3.3.2), malaria (SDG 3.3.3), and hepatitis (SDG 3.3.4). Similarly, SDG 3 constitutes a specific target for NCDs (SDG 3.4) that anticipates reducing by 2030 one-third of premature mortality from NCDs, with target 3.4.1 focusing specifically on reducing the mortality rate attributed to diabetes mellitus, cardiovascular disease, respiratory diseases, and malignant neoplasms that account for 80% of all premature NCD deaths. In the last two decades, substantial global and domestic investments have been made to prevent and manage these diseases, including a range of options for antiretroviral treatment (ART) that helped people with HIV (PWH) live longer with the disease^1–4^. In contrast, the implications of longevity with IDs and their predisposition to chronic NCDs are underexplored and considerably neglected^5–7^.

Some of the most critical targets in SDG 3 could be addressed with the use of digital health interventions where digital and mobile technologies are used and implemented in different ways to support health system needs. These technologies could be telemedicine, artificial intelligence, wearable devices, cloud-based applications, mobile health, big data, and electronic health records. Following the onset of the COVID-19 pandemic, the utility of digital health interventions in combating IDs and NCDs has boldly come into focus^8–10^. For example, there were transformative tools and technologies implemented to prevent, control, diagnose, communicate, manage, and treat diseases during lockdowns and other tumultuous periods. In addition, wearable smartwatch devices have been adapted to detect physiological responses following COVID-19 vaccination^10^ and audio-based digital testing of COVID-19^11^.

There is a growing debate that digital health technologies could assist Africa in realizing the SDG 3 targets and leaving no one behind^12–15^. The growing need for home-grown creativity and international partnerships for co-development of digital health technologies in Africa is promising, which also coincides with the World Health Organization’s (WHO) global strategy on digital health that encourages cross-country collaboration and knowledge transfer in the area^16^. On the other hand, proactive investment in and deployment of digital health technologies intersects with technology characteristics of a given country, including information technology (IT) infrastructure such as access to the internet and mobile phones, electricity, technology intensity, economy, and population literacy. Countries in Africa with weak technology characteristics may fail to deploy and implement digital health technologies successfully. Simultaneously, for efficient use of scarce resources in Africa, it will be important to cultivate synergies with industry for return on investment, market access, entrepreneurship, local production, venture funds, and long-lasting effects.

Drawing on these debates and recognizing the critical role Africa is playing in global health targets and the global economy, there is a dearth of robust information about the ecological facets of digital health interventions in Africa. Important studies and policy insights inform the extent to which digital health interventions have been implemented in Africa and the barriers for the success or lack thereof^17–23^. Full adoption and implementation of digital health technologies in Africa require more training, access to better devices and infrastructure, and more investigations on emerging technologies including artificial intelligence, wearable devices, and big data to provide robust evidence of their potential in Africa^12,15^. However, literature is lacking that characterizes the cross-national dynamics of the macro-level digital health ecosystem in Africa and strategically maps countries of high priority for meaningful and sustainable effects of digital health technologies on the health and well-being of citizens.

Hence, we used international data repositories to characterize and map the digital health ecosystems of all 54 countries in Africa in the context of endemic IDs and NCDs. The research questions examined include: 1) which countries of Africa currently hold relatively better digital infrastructure to drive digital health?; 2) which countries are currently most affected by endemic IDs (HIV, TB, malaria) and NCDs (diabetes mellitus, cardiovascular disease, respiratory diseases, malignant neoplasms)?; 3) what ecological associations exist between technology characteristics and burden of IDs and NCDs in Africa?; and 4) which countries of Africa are high-priority for investment in and deployment of digital health technologies on endemic IDs and NCDs? This study aims to provide strategic guidance for governments about where to prioritize efforts in the implementation of digital health technologies in Africa as key steps to accelerate UN SDG 3 and WHO global strategy on digital health 2020–2025 targets.

## Results

### Technology characteristics

**Fig. 1:**
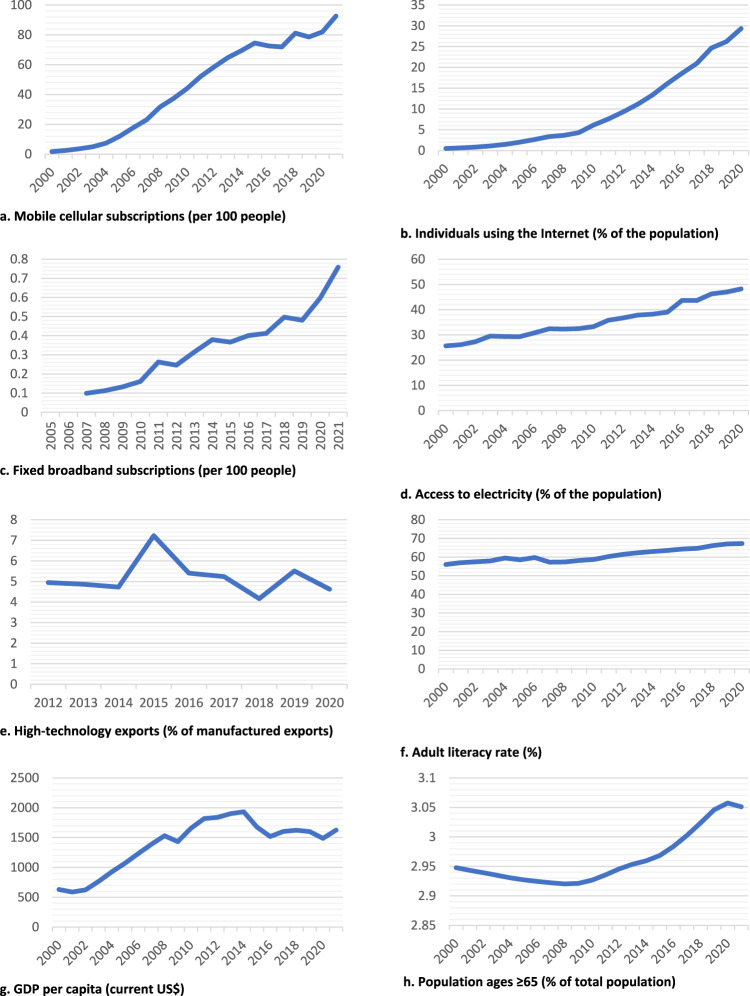
Trends in technology characteristics in Africa, 2000–2021. Trends in technology characteristics in Africa, represented by (**a**) Mobile cellular subscriptions (per 100 people), **b** Individuals using the Internet (% of the population), **c** Fixed broadband subscriptions (per 100 people), **d** Access to electricity (% of the population), **e** High-technology exports (% of manufactured exports), **f** Adult literacy rate (%), **g** GDP per capita (current US$), **h** Population ages ≥ 65 (% of total population).

In 2022, a total estimated population of 1.4 billion people lived in Africa which includes 54 independent countries, with 30% of the population using the internet, 83% having mobile cellular subscriptions, 0.60 per 100 people having fixed broadband subscriptions, and 48.4% having access to electricity. From 2000 to 2021, increases were observed in mobile cellular subscriptions, individuals using the internet, fixed broadband subscriptions, access to electricity, GDP per capita, and adult literacy rates (Fig. 1). The continent had a GDP of US$1.92 trillion with an annual percentage growth of 4.1%, foreign direct investments totaling US$1.7 trillion, and an adult literacy rate of 66%. In 2022, the GDP per capita in Africa reached US$2,150.6, which includes all goods and services produced regardless of their purpose. The overall GDP, with international dollars (INT$) at Purchasing Power Parity (PPP), in 2021, was the highest in Egypt ($1.38 trillion), Nigeria ($1.14 trillion), South Africa ($861.93 billion), Algeria ($532.57 billion), Morocco ($302.77 billion), and Ethiopia ($298.57 billion).

**Fig. 2:**
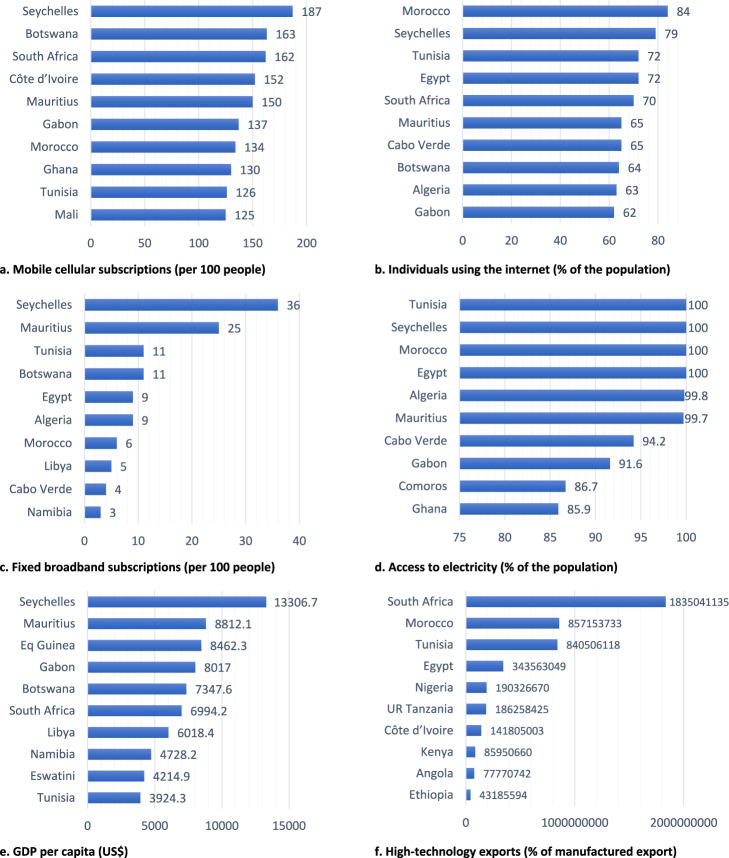
Top 10 countries of Africa with higher technology characteristics, 2021. Top 10 African countries with relatively higher technology characteristics in 2021, represented by (**a**) Mobile cellular subscriptions (per 100 people), **b** Individuals using the Internet (% of the population), **c** Fixed broadband subscriptions (per 100 people), **d** Access to electricity (% of the population), **e** GDP per capita (current US$), **f** High-technology exports (% of manufactured export).

Seychelles is the leading African country in mobile and fixed broadband subscriptions, adult literacy, and GDP per capita. Morocco is the leading in individual internet users’ percentage at 84%; Tunisia, Seychelles, Morocco, and Egypt had 100% electricity access; and South Africa was the leading exporter of high-technology products, more than double that of other African countries. Figure 2 illustrates the top 10 countries by the end of 2021 in each technology characteristics variable.

### Endemic infectious and non-communicable diseases

Of the estimated 38,400,000 PWH at the end of 2021, 25,780,000 (67%) were living in Africa: 20,600,000 (79.9%) in East and Southern Africa, 5,000,000 (19.4%) in West and Central Africa, and 180,000 (0.7%) in Middle East and North Africa. In 2021, 85% of people living with HIV knew their HIV status, 88% of these were accessing ART, and 92% of those accessing ART were virally suppressed. Worldwide, an estimated 9.9 million people fell ill with TB in 2020, where 2,460,000 (25%) of cases were from Africa. Additionally, there were an estimated 241 million cases of malaria worldwide in 2020, of which 95% were from Africa. See comparision of the global versus African burden of endemic IDs in Supplementary information (refer to Supplementary Table 1).

**Fig. 3:**
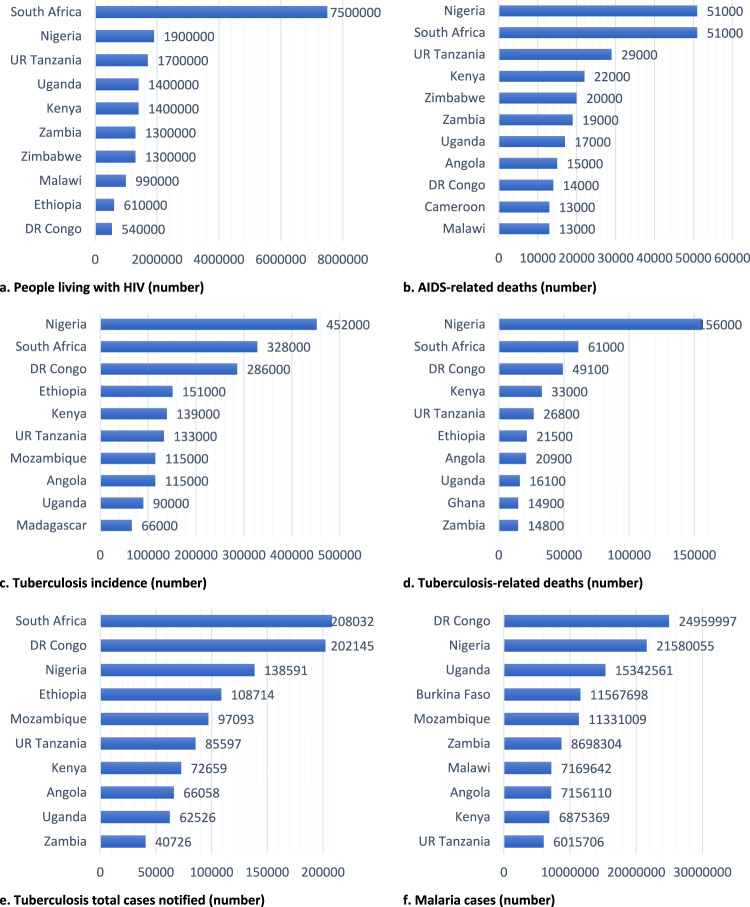
Top 10 countries of Africa with a high burden of endemic infectious diseases, 2021. Top 10 countries of Africa with high HIV, TB, and malaria burden, represented by (**a**) People living with HIV (number), **b** AIDS-related deaths (number), **c** Tuberculosis incidence (number), **d** Tuberculosis-related deaths (number), **e** Tuberculosis total cases notified (number), **f** Malaria cases (number).

Absolute numbers of people living with HIV was the highest in South Africa [7,500,000 (32.4%)], Nigeria [1,900,000 (8.2%)], and the United Republic (UR) of Tanzania [1,700,000 (7.3%)]; TB incidence was the highest in Nigeria [452,000, (17.7%)], South Africa [328,000 (12.9%)], and Democratic Republic of Congo [286,000 (11.2%)]; and malaria cases were the highest in DR Congo [24,959,997 (14.3%)], Nigeria [21,580,255 (12.4%)], and Uganda [15,342,561, (8.8%)]. South Africa had the highest number of laboratory-confirmed MDR/RR TB cases, while Burundi and Mozambique had the highest TB treatment success rates. Tunisia was the only country that covered 100% of its TB treatment funding from domestic sources (Fig. 3).

For NCDs, of the estimated 40.8 million people globally who died due to NCDs in 2019, 7.1% (2,889,945) were from Africa. Africa’s share of global disease-specific deaths was 11% for DM (1,954,067 vs. 215,071), 6.1% for cardiovascular disease (17,900,000 vs. 1,093,577), 4.5% for respiratory diseases (4,136,899 vs. 185,472), and 5.7% for malignant neoplasms (9,296,641 vs. 534,293). The 20-year trends showed that deaths due to NCDs are steadily increasing in Africa, an increase in 789,105 annual deaths compared with 2001 (2,100,840) (refer to Supplementary Fig. 1).

**Fig. 4:**
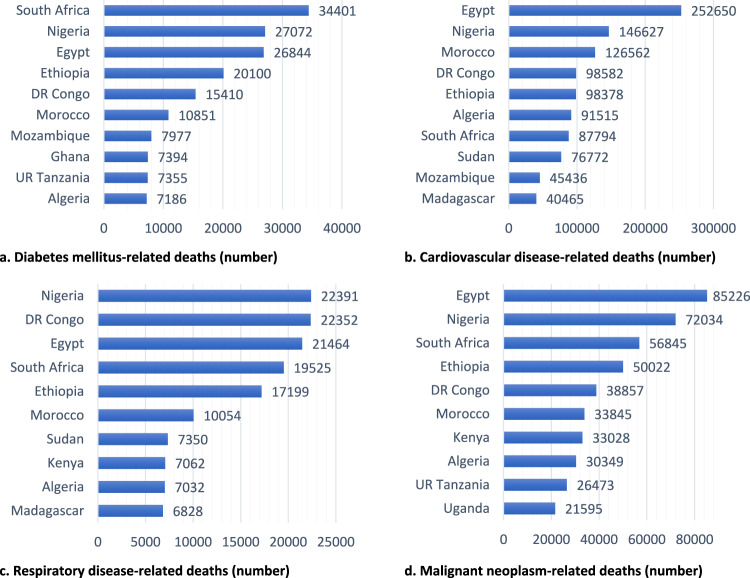
Top 10 countries of Africa with a high burden of endemic non-communicable diseases, 2020. Top 10 African countries with a high burden of endemic non-communicable diseases, represented by the number of deaths attributed to (**a**). diabetes mellitus, **b** Cardiovascular disease. **c** Respiratory disease. **d** Malignant neoplasm.

Egypt had the highest number of cardiovascular disease, malignant neoplasm, and total NCD deaths in Africa. South Africa had the largest number of diabetes-related deaths. Nigeria had the largest number of respiratory disease deaths. Mauritius had the highest prevalence of diabetes among people aged 20 to 79 (Fig. 4).

### Correlation between exposure and outcome variables

Fixed broadband subscription was negatively correlated with TB incidence (*r* = −0.279; *p* = *0.043*), TB-related deaths (*r* = −0.322; *p* = *0.019*), and malaria incidence (*r* = −0.469; *p* = *0.001*). The percentage of the population using the internet was negatively correlated with malaria incidence (*r* = −0.353; *p* = *0.016*). GDP per capita was negatively correlated with TB incidence (*r* = −0.322; *p* = *0.017*), TB-related deaths (*r* = −0.331; *p* = *0.015*), and malaria incidence (*r* = −0.402; *p* = *0.006*). Access to electricity was negatively correlated with HIV prevalence (*r* ≤ −0.291), TB-related deaths (*r* ≤ −0.281), and malaria incidence (*r* = −0.519); *p* < *0.05*. High-technology capacity was negatively correlated with malaria incidence. Mobile subscriptions and adult literacy rate were not correlated with the burden of any infectious diseases (*p* > *0.05*) An increase in high-technology exports was correlated with an increase in the burden of NCDs (*r* ≥ 0.651; *p* < *0.05*), while the rest drivers of digital health variables had no association with any NCDs (Table 1).

**Table 1:** Correlations between exposure (technology characteristics) and outcome (ID and NCD incidence and deaths) variables.

Items	Mobile cellular subscription, per 100 pn.	Individuals using the Internet, %	Fixed broadband subscription, per 100 pn.	Access to electricity, % of pn	High-technology exports, US$	Adult literacy rate, %	GDP per capita, US$
**HIV**							
People living with HIV (*n*)	0.060	−0.077	−0.196	−0.291*	0.454*	0.128	−0.060
AIDS-related deaths (*n*)	0.053	−0.078	−0.234	−0.327*	0.448*	0.084	−0.074
**Tuberculosis**							
Incidence (*n*)	−0.147	−0.214	−0.279*	−0.350*	0.538*	−0.130	−0.322*
Incidence rate (per 100,000 pn)	−0.203	−0.174	−0.241	−0.332*	0.164	0.090	−0.126
TB-related deaths (*n*)	−0.204	−0.259	−0.322*	−0.395*	0.519*	−0.081	−0.331*
Deaths rate (per 100,000 pn.)	−0.135	−0.170	−0.246	−0.281*	−0.199	−0.147	−0.063
**Malaria**							
Cases (*n*)	−0.162	−0.353*	−0.469*	−0.519*	−0.402*	−0.262	−0.402*
**NCDs**							
DM death (*n*)	0.046	0.053	0.002	−0.055	0.711*	−0.075	−0.143
CVD death (*n*)	−0.022	0.005	−0.025	−0.084	0.673*	−0.147	−0.201
RD death (*n*)	−0.030	−0.017	−0.068	−0.110	0.651*	−0.165	−0.244
MN death (*n*)	−0.027	0.002	−0.035	−0.104	0.669*	−0.127	−0.201
Total NCD death (*n*)	−0.022	−0.003	−0.037	−0.098	0.685*	−0.150	−0.206

*r* Spearman’s correlation, *P-value < 0.05, *US$* United States Dollar, *GDP* Gross domestic product, *pn* Population, *DM* Diabetes mellitus, *CVD* Cardiovascular diseases, *RD* Respiratory diseases, *MN* Malignant neoplasms, *NCD* Non-communicable diseases.

### Digital health ecosystem mapping of all 54 countries

Finally, the digital health ecosystem of the 54 countries of Africa was characterized and mapped based on their rank on the WLC model that provides a one-point weighted linear combination of disease burden, mobile/internet subscriptions, and GDP per capita. For NCD-related outcome variables, running a correlation among the five NCD variables, we found a correlation coefficient greater than 0.95, indicating nearly perfect positive colinearity among the NCD outcome variables at a cut-off of > 0.80 (refer to Supplementary Table 2). Hence, in prioritizing and developing a map for NCDs, we considered total NCD deaths as it provided relatively better information about the NCD burden compared to single NCDs. For ID-related outcome variables (refer to Supplementary Table 3), since the correlation coefficient among the two HIV-related outcome variables was 0.967, the number of PWH was considered in the mapping. The correlation among TB-related burdens was greater than 0.88, TB incidence was considered in the mapping. Number of malaria cases was not highly correlated with the other IDs. Therefore, IDs burdens were represented separately by PWH, TB incidence, and number of malaria cases.

For IDs, the highest-priority countries for implementation of digital health technologies were South Africa, Nigeria, and Tanzania for HIV; Nigeria, South Africa, and DROC for TB; and DROC, Nigeria, and Uganda for malaria. For NCDs, Egypt, Nigeria, and Ethiopia were the highest-priority countries for digital health interventions. For details about each specific NCD, refer to Supplementary Table 4. Table 2 lists countries ranked top and least three for digital health interventions on IDs and NCDs.

**Table 2:** Countries in Africa ranked top and least three for digital health ecosystem on endemic infectious and non-communicable diseases.

Countries	South Africa	Nigeria	UR Tanzania	ST & Principe	Comoros	Mozambique
PWH r, n	1 5250731.8	2 1330228.3	3 1190130.8	52 960.7	53 307.5	54 59.8
	Nigeria	South Africa	DR Congo	Cape Verde	ST & Principe	Comoros
TB incidence r, n	1 316628.3	2 230331.8	3 200267.6	52 518.2	53 442.7	54 377.5
	DR Congo	Nigeria	Uganda	Cape Verde	Lesotho	Togo
Malaria cases r, n	1 17472065.5	2 15106266.8	3 10739890.7	52 371.2	53 131.3	54 115.0
	Egypt	Nigeria	Ethiopia	Cape Verde	Comoros	ST & Principe
TNCD death r, n	1 345141.5	2 313665.2	3 190010.9	52 1765.6	53 1739.7	54 665.5

*r* Rank, *n* Number of cases, *PWH* People living with HIV, *DR Congo* Democratic Republic of Congo, *ST & Principle* Sao Tome and Principle, *TB* Tuberculosis, *TNCD* Total non-communicable diseases.

**Fig. 5:**
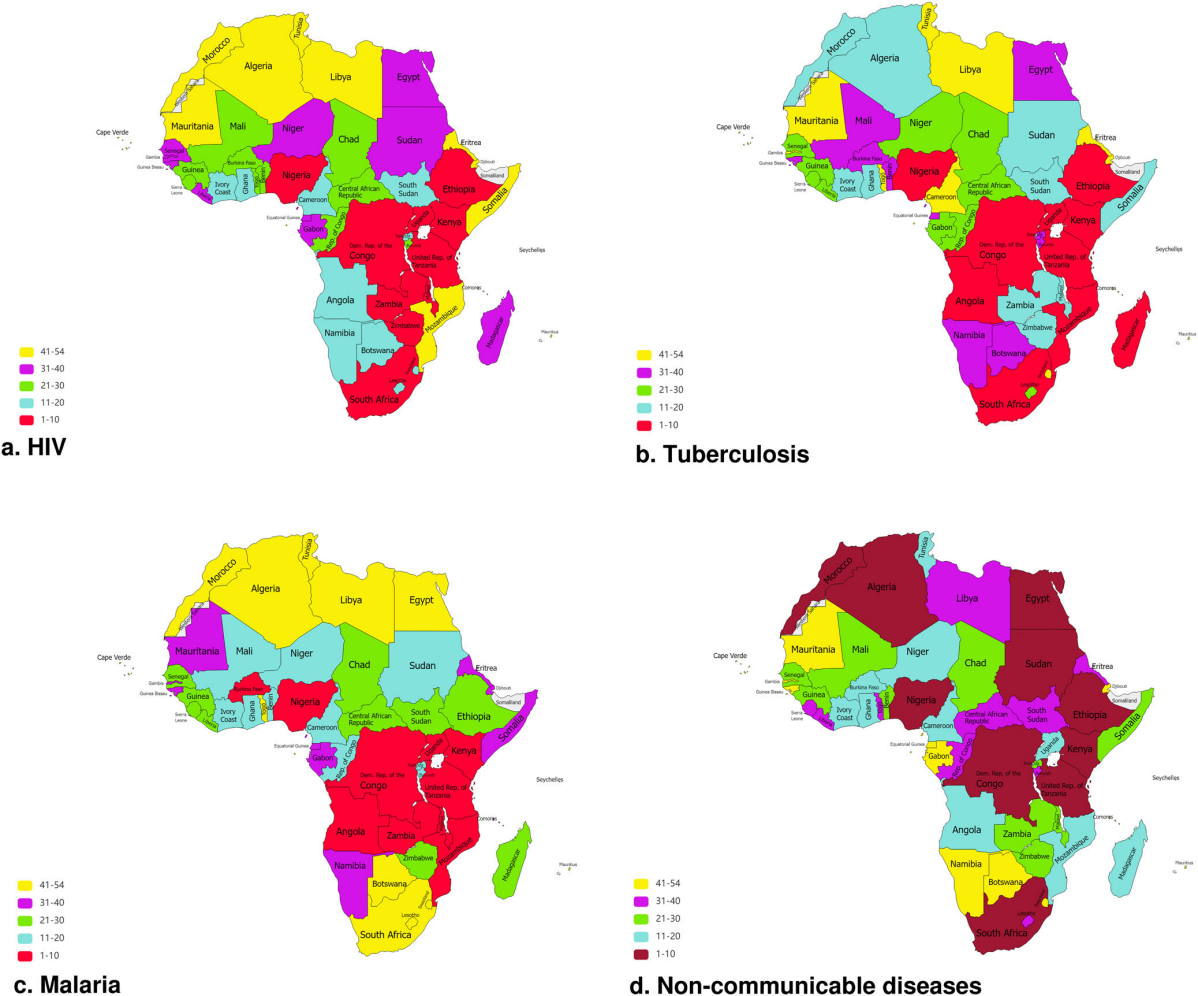
Map of Africa illustrating ranks of countries that should prioritize digital health investments against endemic infectious and non-communicable diseases. Maps illustrating all 54 countries of Africa, based on their ranks in descending order, that should prioritize digital health investments against (**a**) HIV, (**b**) Tuberculosis, (**c**) Malaria, (**d**) Non-communicable diseases across.

Figure 5 mapped the 54 countries of Africa based on their rank for digital health ecosystem on IDs (HIV, TB, malaria) and NCDs, with a higher rank representing countries of high priority.

**Fig. 6:**
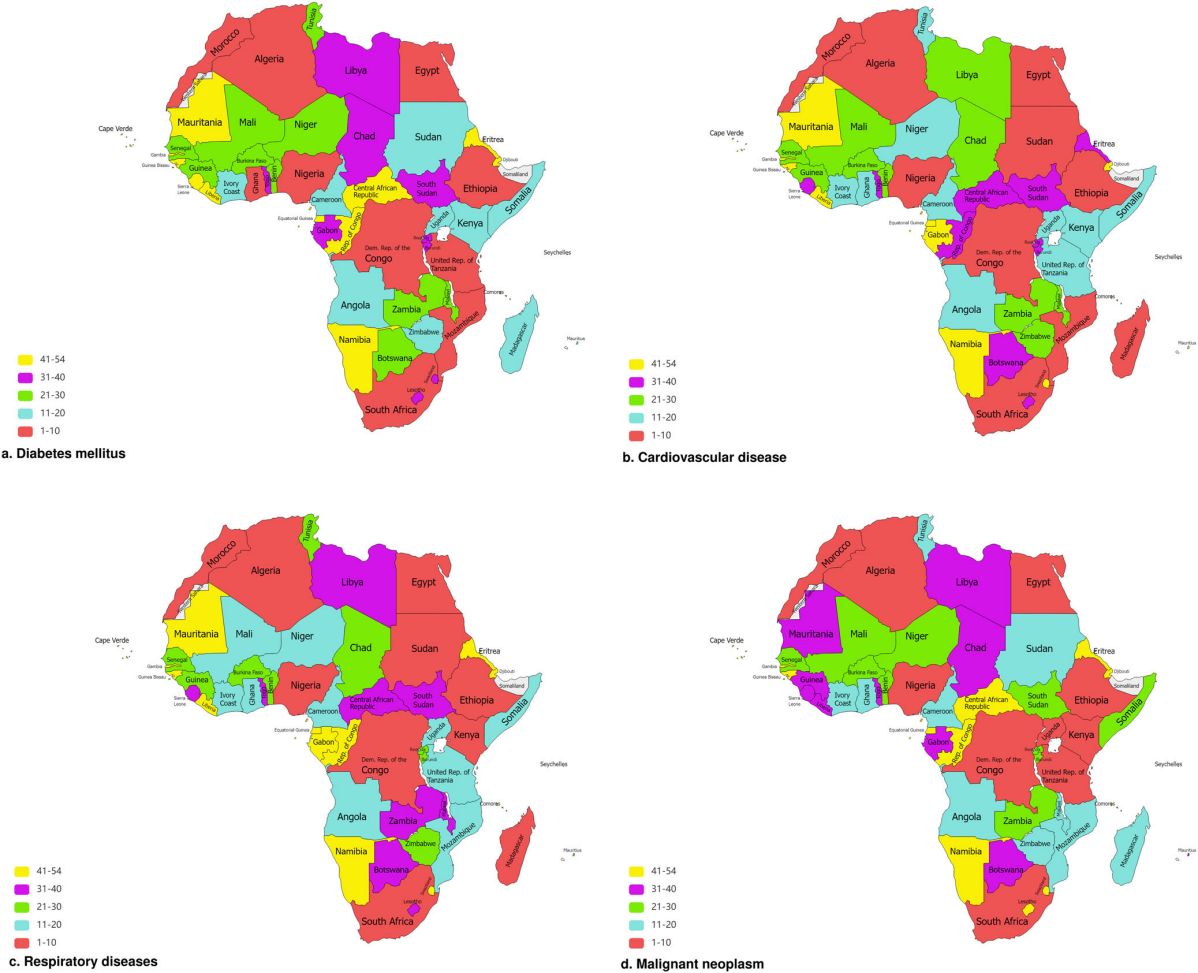
Map of Africa illustrating ranks of countries that should prioritize digital health investments against specific non-communicable diseases. Maps illustrating all 54 countries of Africa, based on their ranks in descending order, that should prioritize digital health investments against (**a**) Diabetes mellitus, (**b**) Cardiovascular disease, (**c**) Respiratory diseases, (**d**) Malignant neoplasm.

Figure 6 mapped the 54 countries of Africa based on their rank for digital health ecosystem of each specific NCD.

## Discussion

Here, we report current digital health ecosystems in Africa and the countries that should prioritize digital health investments against endemic IDs and NCDs. The findings demonstrate that in the last 20 years, critical infrastructure and drivers of digital health interventions have been steadily growing in Africa, including the number of individuals using the internet, mobile cellular subscriptions, fixed broadband subscriptions, high-technology manufacturing, GDP per capita, and adult literacy rate. However, these technology developments varied across countries, with Seychelles, Morocco, Tunisia, South Africa, Mauritius, and Botswana at the top, thus developments concentrate mainly in the Northern part of Africa, followed by Southern Africa. Many African countries were overwhelmed by the dual burden of endemic IDs and NCDs. There were inverse correlations between technology characteristics and IDs, such as fixed broadband subscription and incidence of tuberculosis and malaria, GDP per capita and incidence of tuberculosis and malaria, and access to electricity and prevalence of HIV and malaria. On the WLC ranking, which provides a one-point weighted linear combination of disease burden, existing infrastructure, and economy, the highest-priority countries for implementation of digital health technologies were South Africa, Nigeria, and Tanzania for HIV; Nigeria, South Africa, and Democratic Republic of the Congo (DROC) for tuberculosis; DROC, Nigeria, and Uganda for malaria; and Egypt, Nigeria, and Ethiopia for endemic NCDs (diabetes mellitus, cardiovascular diseases, respiratory diseases, cancer). Countries such as Kenya, Ethiopia, Zambia, Zimbabwe, Angola, Zambia, and Mozambique also were highly affected by endemic IDs but were not on the list of countries with high priority as they had comparably lower technology access and economy.

The returns on digital health investments rely mainly on country-specific demands and enabling environments. In the current study, South Africa, Nigeria, Tanzania, DR Congo, and Uganda were the most prepared and most likely to benefit from digital health interventions on IDs. This finding amplifies the previous report from the WHO Digital Health Atlas platform that listed 12 countries, which exhibited a relatively greater number of digital health projects, including four countries similarly identified by this analysis^24^. However, countries with better performance at the macro-level of the digital health ecosystem may not necessarily experience optimal deployment and implementation of digital health programs in actuality. Previous studies issued cautionary notes on the digital health space in South Africa, including restrictive legislative and regulatory processes^25^, underinvestment^26^, urban-rural divide^27,28^, and disintegration from the multi-sectorial health systems backing SDG 3^29^. South Africa was one of the first countries globally to adopt telehealth/medicine but, because of poor connectivity in rural areas, the program did not flourish^26–28,30,31^. Similarly, in Nigeria, digital health technologies offer profound opportunities to materialize SDG 3 but were concentrated as pilot projects^32^. In general, robust digital health capabilities are lacking in Africa as this is an emerging and consistently evolving technology^33,34^. In addition, even if the supply of digital health technologies exists, it is not clear that it will be adopted optimally by those in the health sector. On the other hand, countries such as Kenya, Ethiopia, Zambia, Zimbabwe, Angola, Zambia, and Mozambique also were highly overwhelmed by endemic IDs but were not on the list for their relatively lower digital infrastructure and economy. Further, by prioritizing certain countries with existing infrastructure, there is a possibility of worsening the existing inequities even if the weight formulation processes were matched by countries with the highest burden. Hence, those countries with a high burden of IDs but lack the needed digital infrastructure should be prioritized for economic development. Poorer countries would most likely invest relatively less in health and hence would be prone to IDs associated with poverty. Therefore mechanisms that broadly uplift economic development are necessary for digital health technologies to become effective.

In the current study, Egypt, South Africa, Nigeria, Morocco, Ethiopia, and DROC were top priority countries for digital health investments in NCDs. They had the highest burden of different types of endemic NCDs and a comparatively better enabling environment for investment in digital health technologies for NCDs. The data also showed that the majority of these countries are ranked the highest in Africa for high-technology exports, which would provide opportunities for in-country digital health technology development and startups. Digital health technologies supporting the management of NCDs are rapidly evolving in the developed world^35–40^, and those African countries highly susceptible to NCDs would need to investigate the effectiveness of such technologies in local contexts to adapt or develop locally and implement sustainably.

In this study, countries that had relatively better economies, IT infrastructure, and electric power supply were less vulnerable to IDs, which was aligned with previous studies that documented a significant association between under-development and infectious diseases^41–43^. This was not the case for NCDs, where the data showed a higher burden of specific NCDs in countries with comparatively better economies and infrastructure, which was comparable with previous findings^44–47^. As technology becomes accessible for a country, this correlates with improved public health practices including water, sanitation and hygiene (WASH), thereby reducing infectious diseases but increasing exposure to unhealthy lifestyles including physical inactivity, unhealthy diet, and other sedentary lifestyles that increase the risk of NCDs^48–51^. Countries in the northern part of Africa including Egypt, Tunisia, Morocco, and Libya are in a relatively better economic and infrastructural situation, but bear a significant share of the NCD burden in Africa.

Africa’s economies are smaller than the economies in other continents of the world and this may hamper Africa’s significant contributions to the digital health ecosystem; however, untapped opportunities exist for strategic actions. Global health initiatives may have significant contributions toward bridging the knowledge and investment gaps, both through technology transfer for local production of digital health technologies and price negotiation for large-scale deployment of the most impactful technologies. The long-term financial and technical support that global health partners such as the United States President’s Emergency Plan for AIDS Relief (PEPFAR) brought to Africa in the fight against endemic IDs^52–57^ could serve as a fertile ground to transform digital health interventions in the area, extend to NCD programs, and avert digital health inequities ahead. This is with full understanding that many of the infrastructure developments alongside digital health technologies remain on the shoulders of local African governments. WHO recommends countries to actively work towards universal health coverage (UHC), and hence, investments in digital health technologies should go beyond vertical disease programs to help strengthen the overall healthcare system. Resilient governance, stewardship, health systems, and societies are all important for wide-scale deployment of digital health technologies through the lens of UHC^16,17,58–60^. Resource-limited countries may initially start with high-burden diseases using tailored digital health technologies within existing resources. Then over time, these same technologies could be leveraged and adapted to respond to other health priorities thereby creating broader access to digital health. This is precisely how countries used HIV-specific resources, technologies, programs, services, and systems to address other priorities such as Ebola, COVID-19, measles, and now NCDs^53,61–63^.

This study uses rigorous methods, addressing all 54 countries of Africa, to unlock information gaps in the digital health ecosystem in the context of IDs and NCDs of global importance in Africa. However, this study has some limitations. The study explored the macro-level digital health ecosystem to understand the broader forces that impact investments in digital health technologies in Africa, hence, insights from micro- and meso-level analyses including patient- and provider-reported usability and satisfaction results are also needed to understand individual forces that could be observed in the implementation of a specific digital health technology. The study mapped the digital health ecosystem and underlying facets in the context of endemic IDs and NCDs, and thus may not necessarily address non-endemic diseases.

In conclusion, this study provides substantial insight into where governments should prioritize efforts in digital health interventions in Africa as an important accelerator of the UN SDG 3 targets by 2030 and the WHO global strategy on digital health 2020–2025. Many African countries were overwhelmed by a double burden of infectious and non-communicable diseases. Conversely, in the last 20 years, infrastructure important to digital health interventions hase been steadily growing in Africa, including the number of individuals using the internet, mobile cellular subscriptions, fixed broadband subscriptions, high-technology exports, adult literacy rate, and GDP per capita. Digital health technology investments in Africa, including clinical utility trials, require preliminary analyses of the enabling environments to bring about sustainable health and economic returns. Equally, African countries with a high burden of endemic diseases but lacking digital infrastructure should be prioritized for economic development not to worsen existing inequities. Building digital infrastructure should be a key part of economic development programs in countries with high disease burdens to ensure more equitable health outcomes. Though infrastructure developments alongside digital health interventions remain on the shoulders of governments, global health initiatives can cultivate digital health interventions substantially by bridging knowledge and investment gaps, both through technology transfer for local production and negotiation of prices for large-scale deployment of the most impactful digital health technologies.

## Methods

### Design and data sources

For 54 countries across Africa, we conducted a cross-national ecological analysis using data collected from four credible open-access sources: The World Bank Open Data^64^, the WHO Global Health Observatory^65^, Africa United Nations Data for Development of the United Nations Economic Commission for Africa^66^, and the Global Data on HIV Epidemiology and Response of the Joint United Nations Programme on HIV/AIDS (UNAIDS)^67^. A minimum of 20 years of consecutive annual data, from January 2000 to September 2022 (latest date available), was extracted for 38 independent variables on technology characteristics and incidence or mortality of endemic IDs and NCDs. The World Bank Open Data consists of 5,443 global development datasets and the WHO Global Health Observatory data repository provides credible data on over 1000 indicators on priority health topics. The Africa UN Data for Development platform provides a regional online portal to bring together all African countries with high-quality data and statistical progress on SDG 3030 as a one-stop-shop repository. The UNAIDS provides the world’s most credible and large-scale datasets on epidemiology, program coverage, and finance relevant to HIV/AIDS.

We adapted the WHO digital health interventions framework^68^ to provide a conceptual framework that helps operationalize and interpret the findings of this study. Our underlying focus was to study if a country’s maturity of the digital ecosystem comprising the technology infrastructure and economy (i.e., the exposure) has a critical influence on the relevance and impact of digital health interventions in reducing the incidence and mortality of endemic IDs and NCDs (i.e., the outcome).

### Variables

Using the World Bank Open Data, 20-year data were collected for each African country on nine key exposure variables including economic size, IT infrastructure, electricity infrastructure, technology intensity, and population literacy that drive digital health implementation. Using the WHO Global Health Observatory data repository, data were collected for each African country on 17 key variables on endemic IDs and NCDs. Using the Africa UN Data for Development portal, analyses were made on key SDG 3 trends and progress so far in Africa, and data were used to understand the progress toward SDG 3 in Africa. The UNAIDS database was used to analyze trends in key HIV/AIDS indicators and to compare the burden of HIV/AIDS in Africa against the globe and among sub-regions within Africa based on the latest (2022) data as available. These variables were selected following a review of the complete list of variables in each database. Those that provide the best fit to the study of interest were pooled in line with WHO’s digital health interventions framework and WHO’s definitions of endemic IDs and NCDs. The variables pooled from the four database platforms are summarized in Supplementary Table 5.

### Data analysis

Spearman’s rank correlation coefficients were used to characterize the ecological associations between exposures (technology characteristics) and outcomes (incidence or mortality of endemic IDs and NCDs).

The variables used to define the exposure were mobile cellular subscriptions (per 100 people); individuals using the internet (% of the population); fixed broadband subscriptions (per 100 people); access to electricity (% of the population); high-technology exports (US$); adult literacy rate (%); and GDP per capita (US$).

The variables used to define the outcome had two categories: IDs and NCDs. Thus, outcome variables included incidence or mortality of IDs (HIV, TB, malaria) and NCDs (diabetes mellitus, cardiovascular diseases, malignant neoplasm, respiratory diseases, the four NCDs in total). Additional programmatic aspects including treatment success, drug resistance, and financing were included. We used descriptive statistics to characterize the variables.

We conducted a colinearity test at a cut-off value of Spearman’s rank correlation coefficient of > 0.80 to identify and exclude variables providing similar information and minimize the number of variables accordingly. Descriptive analyses were conducted to display in bar charts the top ten countries for each exposure and outcome variable and to show, using line charts, the patterns over 20 years of the variables. Incidence and mortality of outcome variables were compared between Africa and globally as well as between sub-regions within Africa which were East and Southern Africa, West and Central Africa, and the Middle East and North Africa. The findings were summarized in tables.

A weighted linear combination (WLC) model^69^ was used as the decision rule, combining disease burden, technology access, and GDP per capita, to explain, rank, and map the digital health ecosystem of a given country. Informed by the WHO digital health framework^68^, a counterfactual scenario was formulated that combined three attributes from the exposure and outcome variables: 1) countries with the highest burden of infectious or NCDs should have priority for digital health technologies in line with the specific disease and the related digital health intervention; 2) all countries with the highest burden of IDs or NCDs may not have favorable access to the internet or mobile services for successful digital health interventions, and hence the high-burden countries would need to also have relatively high access to the internet or mobile phone subscription rate depending on the nature of the digital health technology; and 3) all countries with the highest burdens of IDs or NCDs and better access to the internet or mobile subscriptions may not have the economy to import or locally produce digital health technologies and hence would also need to have a competitive economy. Hence, disease burden (incidence or mortality of IDs and NCDs), digital infrastructure (access to the internet or mobile phone subscription), and economy (GDP per capita) were identified as the main attributes defining the digital health ecosystem of a given country. Based on the WLC model, weights were determined by an expert group using multiple attribute decision-making (MADM)^69^. With these, a one-point linear combination was developed: [0.7*incidence/mortality of the disease] + [0.2*mobile/internet subscriptions] + [0.1*country GDP per capita] of a given country. Using the WLC procedure, we then ranked all 54 countries according to their overall values, and for each ID and NCD and considering colinearity. We developed map charts using an online map creation platform^70^ to illustrate countries based on their ranks and in descending order.

### Reporting summary

Further information on research design is available in the Nature Research Reporting Summary linked to this article.

## Supplementary information

Reporting Summary

Supplementary Information

## Data Availability

The data that support the findings of this study were derived from the following open-access public domain resources: The World Bank Open Data, the WHO Global Health Observatory, Africa United Nations Data for Development of the United Nations Economic Commission for Africa, and the Global Data on HIV Epidemiology and Response of the UNAIDS.
