# Supplementary material for: Mapping digital health ecosystems in Africa in the context of endemic infectious and non-communicable diseases
Source: NPJ Digit Med. 2023 May 26;6:97. doi: 10.1038/s41746-023-00839-2 (PMC10213589; doi:10.1038/s41746-023-00839-2)
Supplement: Supplementary file 2 — Supplementary Information [file 41746_2023_839_MOESM2_ESM.pdf]

Supplementary information

Mapping the digital health ecosystem in Africa in the context of endemic infectious and non-communicable diseases: A cross-national analysis of 54 countries

Supplementary Table1. Comparison of the global versus Africa burden of endemic infectious diseases, 2021. DR: drug-resistant; MDR: multidrug-resistant; US$: United States Dollar.

Infectious disease	Indicator	Global burden	African share
**HIV**	People living with HIV	38.4 million	25.78 million (67.1%)
	On antiretroviral therapy	28.7 million	20.19 million (70.3%)
	Estimated incidence	1.5 million	874,000 (58.3%)
	HIV-related deaths	650,000	425,100 (65.4%)
	Pre-treatment prevalence of DR-HIV	156,982	18,393 (11.7%)
	Nucleoside reverse-transcriptase inhibitor resistance	5.4%	6.1% (113%)
	Efavirenz or nevirapine resistance	12.9%	15.4% (119.4%)
	Atazanavir, darunavir, lopinavir/ritonavir resistance	0.4%	0.3% (75%)
	Integrase strand-transfer inhibitor resist.	0.6%	0.1% (16.7%)
	Available annual funding 2021 (US$)	21.4 billion	12.2 billion (57%)
**Tuberculosis**	Incidence	9.87 million	2.46 million (25%)
	Notified	6,154,338	1,405,329 (22.8%)
	Mortality	1,494,000	549,000 (36.7%)
	HIV-TB incidence	787,000	579,000 (73.6%)
	Confirmed MDR/RR-TB	156,982	18,393 (11.7%)
	Available annual funding, 2021 (US$)	4,877,000,000	758,770,000 (15.5%)
**Malaria**	Incidence	241 million	228 million (94.6%)
	Deaths	627,000	602,000 (96%)
	Available annual funding, 2021 (US$)	3.3 billion	2.6 billion (79%)

Supplementary Table 2. Co-linearity between countries' mortality attributed to endemic non-communicable diseases. r: Spearman's correlation; * P-value <0.05; DM: Diabetes mellitus; CVD: cardiovascular diseases; RD: respiratory diseases; MN: malignant neoplasms; NCD: non-communicable diseases

Items	DM death (n)	CVD death (n)	RD death (n).	MN death (n)	Total NCD death (n)
DM death (n)	1				
CVD death (n)	.962*	1			
RD death (n)	.967*	.976*	1		
MN death (n)	.957*	.969*	.957*	1	
Total NCD death (n)	.974*	.993*	.980*	.983*	1

Supplementary Table 3. Collinearity between countries' burden of endemic infectious diseases. r: Spearman's correlation; * *P-value* <0.05; DR: drug-resistant; MDR: multidrug-resistant; pn: population

Items	People living with HIV	AIDS-related deaths	TB Incidence (n)	TB Incidence rate (per 100,000 pn)	TB-related deaths (n)	TB Deaths rate (per 100,000 pn.)	TB Treatment coverage (%)	TB Total cases notified	Lab-confirmed MDR/RR-TB	Treatment success rate (%)	Malaria Cases (n)
**HIV**											
People living with HIV	1										
AIDS-related deaths	.967*	1									
**Tuberculosis**											
Incidence (n)	.658*	.688*	1								
Incidence rate (per 100,000 pn)	.453*	.508*	.473*	1							
TB-related deaths (n)	.747*	.777*	.942*	.571*	1						
Deaths rate (per 100,000 pn.)	.499*	.532*	.408*	.941*	.563*	1					
Treatment coverage (%)	-.023	-.056	-.016	-.353*	-.168	-.567*	1				
Total cases notified	.660*	.683*	.936*	.360*	.911*	.286*	.133	1			
Lab-confirmed MDR/RR-TB	.707*	.722*	.882*	.562*	.889*	.520*	-.034	.852*	1		
Treatment success rate (%)	.089	-.003	.273*	-.168	.180	-.223	.438*	.390*	.264	1	
**Malaria**											
Cases (n)	.557*	.566*	.593*	.004	.624*	-.006	.128	.591*	531*	.214	1

Supplementary Table 4. Countries in Africa ranked top and least three for digital health ecosystem on endemic non-communicable diseases. r: rank; n: number of cases; DR Congo: Democratic Republic of Congo; ST and Principle: Sao Tome and Principle; DM: Diabetes mellitus; CVD: cardiovascular diseases; RD: respiratory diseases; MN: malignant neoplasms

	South Africa	Egypt	Nigeria	Gambia	Comoros	ST & Principe
DM death	1	2	3	52	53	54
r, n	24812.5	19197.0	19178.7	364.1	284.1	279.0
	Egypt	Nigeria	Morocco	Cape Verde	Comoros	ST & Principe
CVD death	1	2	3	52	.53	54
r, n	177261.2	102867.2	88969.9	969.8	801.4	428.4
	Nigeria	DR Congo	Egypt	Guinea-Bissau	ST & Principe	Comoros
RD death	1	2	3	52	53	54
r, n	15902.0	15714.0	15431.0	355.4	307.9	266.1
	Egypt	Nigeria	South Africa	Gambia	Comoros	ST & Principe
MN death	1	2	3	52	53	54
r, n	60064.4	50652.1	40523.3	581.3	430.2	322.8

Supplementary Table 5: Databases and variables used for measures of disease burdens and the macro-level drivers of digital health in Africa. TB: tuberculosis; NCD: non-communicable diseases; UN ECA; United Nations Economic Commission for Africa; GDP: Gross domestic product; US$: United States dollar

**WHO Global Health Observatory data**	**World Bank Open Data**
- HIV: Number of People Living With HIV (Number);	- Mobile Cellular Subscriptions (per 100 people);
- TB Total Incidence (Number);	- Individuals Using the Internet (% of the population);
- TB Total Incidence (Rate per 100 000 population);	- Fixed Broadband Subscriptions (per 100 people);
- TB Mortality (Number);	- Access to Electricity (% of the population);
- TB Mortality (Rate per 100 000 population);	- High-Technology Exports (% of manufactured exports);
- TB Treatment Coverage (% notified/estimated incidence);	- Adult literacy rate (%);
- TB Total Cases Notified (Number);	- GDP per Capita (US$);
- TB-MDR/RR Laboratory-confirmed cases (Number);	- Population Growth (Annual %); and
- TB Treatment Success Rate (%);	- Population Ages 65 and Above (% of Total Population).
- TB Financing from Domestic Sources (%);	
- Malaria Confirmed Cases (Number)	
- Diabetes mellitus (DM): Number of Deaths Attributed to DM (Number);	
- DM: Prevalence (% of Population Ages 20-79)	
- Cardiovascular diseases (CVD): Number of Deaths Attributed to CVD (Number);	
- Respiratory Diseases (RD): Number of Deaths Attributed to RD (Number);	
- Malignant Neoplasm (MN): Number of Deaths Attributed to MN (Number);	
- NCDs: Number of Deaths Attributed to the four NCDs in Total (Number)	
**Joint United Nations Programme on HIV/AIDS**	**UN ECA, Africa United Nations Data for Development**
- Total number of PWH	- Trends in GDP per Capita
- Total number of individuals on ART	- Trends in SDG 3
- Estimated incidence of HIV infections	
- HIV-related deaths	
- Pre-treatment prevalence of Drug Resistant-HIV	
- Percentage of individuals with nucleoside reverse-transcriptase inhibitor (NNRTI) resistance	
- Percentage of individuals with efavirenz or nevirapine resistance	
- Percentage of individuals with atazanavir, darunavir, lopinavir/ritonavir resistance	
- Percentage of individuals with integrase strand-transfer inhibitor resistance	
- Available annual funding for HIV in 2021 (US$)	

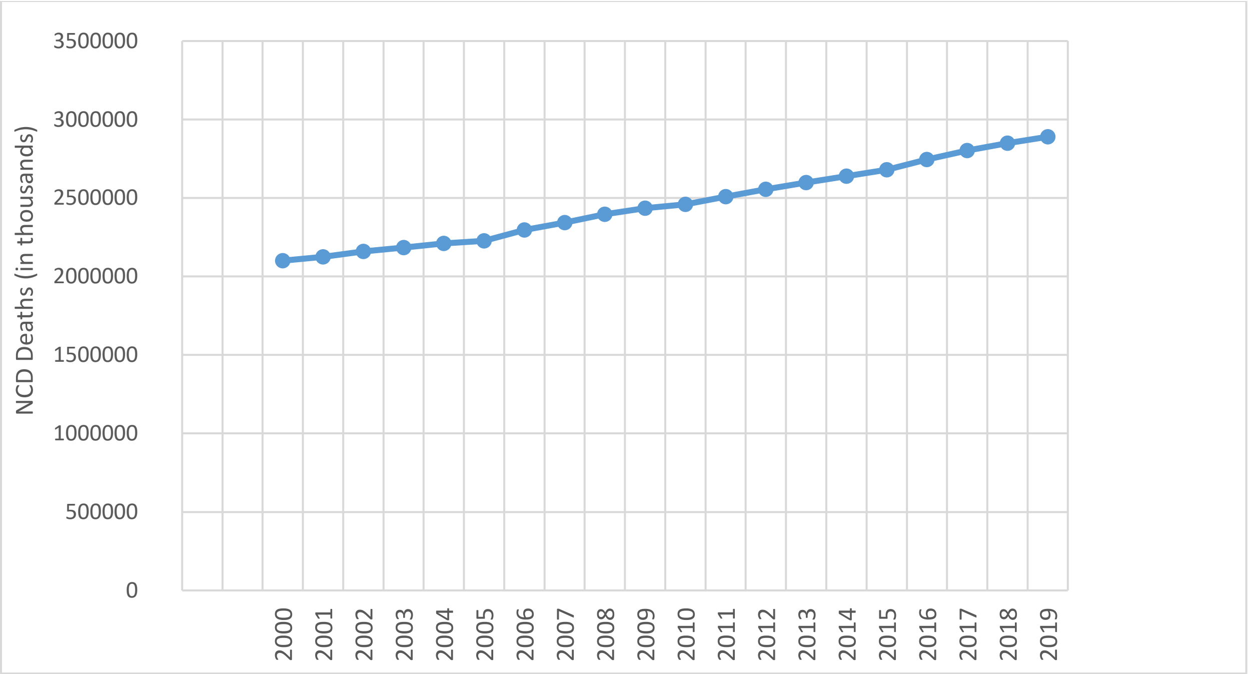

Supplementary Fig. 1: **NCD Deaths in Africa, 2000 – 2019**. Trends in the year 2000 to 2019 of deaths attributed to endemic non-communicable diseases in Africa
